# The Significance of the 2013 Nobel Prize in Chemistry and the Challenges Ahead

**DOI:** 10.1371/journal.pcbi.1003423

**Published:** 2014-01-02

**Authors:** Ruth Nussinov

**Affiliations:** 1 Cancer and Inflammation Program, Leidos Biomedical Research, Inc., Frederick National Laboratory for Cancer Research, National Cancer Institute, Frederick, Maryland, United States of America; 2 Sackler Institute of Molecular Medicine, Department of Human Genetics and Molecular Medicine, Sackler School of Medicine, Tel Aviv University, Tel Aviv, Israel

Last week, the 2013 Nobel Prize in Chemistry was awarded to Martin Karplus, Michael Levitt, and Arieh Warshal for “the development of multiscale models for complex chemical systems”. As the Royal Swedish Academy of Sciences noted, “Chemists used to create models of molecules using plastic balls and sticks. Today, the modelling is carried out in computers. In the 1970s, Martin Karplus, Michael Levitt and Arieh Warshel laid the foundation for the powerful programs that are used to understand and predict chemical processes. Computer models mirroring real life have become crucial for most advances made in chemistry today.” Furthermore, “Today the computer is just as important a tool for chemists as the test tube. Simulations are so realistic that they predict the outcome of traditional experiments.” [1]

This event is a milestone for the broad community that *PLOS Computational Biology* represents. Along with Philip E. Bourne, the Founding Editor-in-Chief, and our Editorial Board, which proudly lists Michael Levitt among its members, I extend the warmest congratulations to the winners. Beyond the specific, personal scientific achievements that have already been widely discussed, we must consider the more general and broader context of this unique prize. Here, I would like to present this Nobel Prize within this framework, emphasizing its magnitude and far-reaching implications not only for computational biology, but for the biological community at large.

In recent decades, molecular biology has progressed by leaps and bounds. Huge technological advances have taken place in sequencing; in mapping structure and dynamics via electron microscopy (EM), X-ray, and nuclear magnetic resonance (NMR); in manipulating imaging of nuclei and cells; in sequencing single biomolecules; and more. These have led to fundamental new insights; biology and medicine have soared to new heights with the DNA double helix providing the molecular basis for genetics and Darwinism. Many steps were required to identify and untangle DNA-RNA-protein sequence-structure-function and reverse transcription processes; RNA enzymes; the importance of key multi-partnered scaffolding molecules under normal physiological conditions and in disease; their structures, mutations, and the principles and mechanisms of their dynamic regulation; and other landmark developments. These involved technological breakthroughs and greater understanding of the specific mechanisms involved. Most of the Nobel prizes in chemistry and medicine in recent years have been awarded at these junctures.

Vast amounts of information on sequences and structures are yet to be explained and pose a challenge for computational biology. Recently, this has been compounded by interdisciplinary studies of the nervous system, posing questions such as how it is structured, how it develops, how it works, the mechanisms of signal processing, and more, all at multiple levels, ranging from the molecular and cellular levels to the systems and cognitive levels. Thus, even if we gain in-depth insight into static properties such as the genomic data and structural snapshots of proteins (DNA and RNA) at different levels of resolution, the truly monumental challenge of understanding their dynamics still looms ahead. And eventually, it is the dynamics of molecules that provides the basis for cells, tissues, and organisms' development and work.

The systems in question operate at all scales: force fields and free energy landscapes relevant for protein folding and function, large complexes, biomolecular recognition involving proteins, DNA, RNA, lipids, post-translational (and DNA) modifications, and interactions with small molecules. On a larger scale we see cellular locomotion, cell division and trafficking, and cell-cell recognition. Furthermore, beyond these lurks the working of the complex cell as a cohesive unit: the cellular network controls metabolism and regulation, intra- and inter-cellular signaling, and the neural circuits of nerve cells, where the activity of one cell directly influences many others. All are dynamic, all change with the cellular environment, and all present a daunting challenge. The relevant timescales range from femtosecond for simple chemical reactions to the eons of evolution; however, all operate with the same underlying physical principles of conformational variability and selection.

At each timescale and corresponding physical size we strive to identify the relevant moving parts and degrees of freedom and to formulate effective—though often approximate—rules for their mutual interactions and resulting motion. Solving, understanding, and computing the dynamic behavior at any given scale is of great interest in its own right and provides approximate dynamical input for the next scale, which is one rung above it. Only at the lowest, most basic scale of individual atoms and electrons are the dynamical rules (electrostatics and Schrödinger's equation) completely well defined. And the all-important work cited by the Nobel Prize Committee and which is carried out by our community is roughly at the first/second level, making it of fundamental importance.

This Nobel Prize is the first given to work in computational biology, indicating that the field has matured and is on a par with experimental biology. It may also be the very first prize given in *any* area of the exact sciences for calculations. What is different in the present case? I believe that the answer is simple: the present calculations are of much greater interest to a much broader community. In endeavoring to imitate the basic processes of life in silico, great strides are being made toward understanding the secret of life. Computational biology, and simulations, for which Martin Karplus, Michael Levitt, and Arieh Warshal shared the Nobel Prize, can carry the torch leading the sciences to decipher the elemental processes and help alleviate human suffering.

What are the challenges ahead? Are simulations with timescales of microseconds, milliseconds, or beyond, under the current force field framework, capable of producing results in agreement with experiments also for large and complex proteins like membrane receptors? Do the challenges also lie in the type of questions which are asked, for which such long timescale simulations can be useful in providing answers? Or is it the biology behind the questions that is also the key? Ultimately, as in experimental biology which also exploits methods and machines, it is likely to be all of the above. Computations are our treasured tool; they are not our aim. Merely running long molecular dynamics trajectories is unlikely to advance science.

*PLOS Computational Biology* joins the International Society of Computational Biology (ISCB) and our computational biology community in congratulating the awardees and celebrating this momentous event.

This Editorial was first published as a blog post on PLOS Biologue on October 18, 2013.
